# Characterization of Exosporium Layer Variability of *Clostridioides difficile* Spores in the Epidemically Relevant Strain R20291

**DOI:** 10.3389/fmicb.2020.01345

**Published:** 2020-07-02

**Authors:** Marjorie Pizarro-Guajardo, Paulina Calderón-Romero, Alba Romero-Rodríguez, Daniel Paredes-Sabja

**Affiliations:** ^1^Microbiota-Host Interactions and Clostridia Research Group, Facultad de Ciencias de la Vida, Universidad Andrés Bello, Santiago, Chile; ^2^Millennium Nucleus in the Biology of the Intestinal Microbiota, Santiago, Chile

**Keywords:** clostridium difficile, spores, exosporium layer, exosporium layer variability, electron microscope analysis

## Abstract

*Clostridioides difficile* is a Gram-positive anaerobic intestinal pathogenic bacterium and the causative agent of antibiotic-associated diarrhea. *C. difficile* spore is a dormant state which acts as a vehicle of transmission and infection. In *C. difficile* spores, the outermost exosporium layer is the first barrier of interaction with the host and should carry spore ligands involved in spore-host interactions. *C. difficile* forms two types of spores (i.e., thin and thick exosporium layers). In this communication, we contribute to understand several biological aspects of these two exosporium morphotypes. By transmission electron microscopy, we demonstrate that both exosporium morphotypes appear simultaneously during sporulation and that spore-coat laminations are formed under anaerobic conditions. Nycodenz density-gradient allows enrichment of spores with a thick-exosporium layer morphotype and presence of polar appendage. Using translational fluorescent fusions with exosporium proteins BclA3, CdeA, CdeC, and CdeM as well as with several spore coat proteins, we observed that expression intensity and distribution of SNAP-translational fusions in R20291 strain is highly heterogeneous. Electron micrographs demonstrate that multicopy expression of CdeC, but not CdeM, SNAP translational fusion, increases the abundance of the thick exosporium morphotype. Collectively, these results raise further questions on how these distinctive exosporium morphotypes are made during spore formation.

## Introduction

*Clostridioides difficile* is the leading cause of nosocomial antibiotic-associated diarrhea (Rupnik et al., 2009; Martin et al., 2016). Recurrence of CDI is a major problem with an incidence of 25–65% of patients, associated to increased severity of symptoms and time in hospital settings (Lessa et al., 2015). The main factor involved in the recurrence of CDI is the formation of metabolically dormant spores during the infection (Deakin et al., 2012). These newly formed spores are essential for the persistence of *C. difficile* in the host and the transmission of the disease to a new susceptible host (Deakin et al., 2012). Sporulation is initiated by an asymmetric cell division that leads to the formation of the mother cell and the incipient forespore (Fimlaid et al., 2013). Later, the mother cell engulfs forespore and mediates the assembly of structural layers, including spore peptidoglycan cortex, inner and outer coat, and exosporium. Finally, the mother cell lyses and releases the mature spore (Fimlaid et al., 2013). Sporulation in *C. difficile* is asynchronous, therefore at a given time, multiple stages of development can be found (Pereira et al., 2013), thus hampering the study of synchronized cultures.

The outermost surface of *C. difficile* spores is thought to play an important role in host-spore interaction by ligands involved in interactions between spore and host cellular receptors (Paredes-Sabja and Sarker, 2012; Mora-Uribe et al., 2016; Calderón-Romero et al., 2018). Recent evidence indicates that *C. difficile* produces spores with two distinctive morphotypes: that is, an exosporium layer with a thin electron dense layer, and an exosporium layer with a thick electron dense layer forming prominent bumps (Pizarro-Guajardo et al., 2016a, b). By sonication and trypsin digestion of the spore surface, enriched fractions of exosporium peptides revealed the presence of several proteins which were shown to be unique for this outer most layer (Díaz-González et al., 2015). These include, three cysteine-rich proteins (CdeA, CdeC, and CdeM) and three collagen-like proteins (BclA1, BclA2, and BclA3) (Pizarro-Guajardo et al., 2014; Díaz-González et al., 2015). Some spore coat proteins, including CotA, CotB, CotD, and CotE were also observed, most likely as part of the spore-coat surface that interacts with the exosporium layer (Díaz-González et al., 2015). The spore coat and exosporium layer are formed during late stages of sporulation, under the control of sigma factors SigE and SigK (Fimlaid et al., 2013), which regulate the expression of the aforementioned proteins. However, the mechanisms that govern exosporium assembly remain unclear.

To date, two morphogenetic cysteine-rich proteins, CdeC and CdeM, have been identified as essential for the correct assembly of the exosporium layer (Barra-Carrasco et al., 2013; Antunes et al., 2018; Calderón-Romero et al., 2018). Both are required for the location of several spore-coat and exosporium proteins to the outer spore outer layer (Calderón-Romero et al., 2018) and the absence of CdeM seems to be involved in the conformation of electron dense material in the spore surface (Antunes et al., 2018). How these proteins contribute to the variability observed in the outermost layer of *C. difficile* spores remains unclear. In this work, we explore new features in exosporium layer variability. We used transmission electron microscopy to demonstrate that thick and thin exosporium spores are produced simultaneously during sporulation of *C. difficile*. By a density gradient, spores can be separated and an enriched fraction in thick-exosporium spores and appendage containing spores can be obtained. By the use of a fluorescence reporter, we observed high heterogeneity in fluorescence intensity and distribution of protein-SNAP fusions. Notably, CdeC-, but not CdeM-SNAP translational fusion, leads to an increase in the relative abundance of thick exosporium spores and exacerbated thick exosporium layer. Collectively, these results provide more evidence of the high variability of *C. difficile* spore outermost layer.

## Materials and Methods

### Preparation of Sporulating Cultures and Purified Spores of *C. difficile* R20291

**FIGURE 1:**
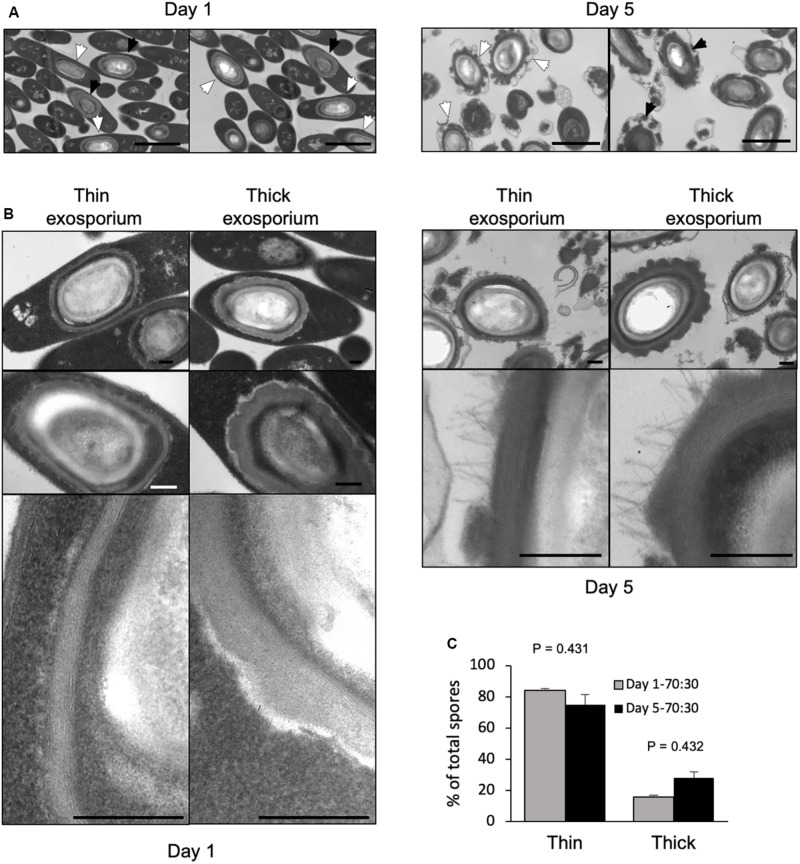
Transmission electron micrographs of 1-day- and 5-day-old sporulating cultures in 70:30 agar plates. **(A)** Sporulating cultures in 70:30 medium harvested after 1 day (left) and 5 days (right) of growth in 70:30 medium. Cultures were fixed under anaerobic conditions prior to processing for transmission electron micrograph analysis. White arrow: thin exosporium spore, Black arrow: thick exosporium spore. Bar scale: 2 μm. **(B)** Ultrastructural analysis of the exosporium layer. Thin and thick exosporium morphotype spores evidenced at day 1 (left panel) and day 5 (right panel). Scale bar: 200 nm. **(C)** Percentage of thin and thick exosporium at day 1 (gray bar) and day 5 culture (black bar). Bar indicates standard error obtained with three different batch of culture fixed, processed and analyzed separately. *N* = 50 spores. *P*-values indicated.

To obtain sporulating cultures, *C. difficile* strain R20291 was routinely grown under anaerobic conditions on BHIS broth (3.7% Brain Heart Infusion supplemented with 0.5% yeast extract, 1% cysteine). 16 h cultures were diluted to 1:500 dilution and 100 μl were plated onto 70:30 (63 g Bacto peptone, 3.5 g protease peptone, 11.1 g BHI medium, 1.5 g yeast extract, 1.06 g Tris, 0.7 g NH_4_SO_4_, 15 g agar per litter) or TY (3% Trypticase Soy-0.5% yeast extract) agar plate and incubated at 37°C for 1 or 5 days, accordingly (see Figure 1 and Supplementary S1), and for 7 days for spore purification. After incubation, plates were scraped up with 1 ml of ice-cold sterile water. Sporulating cultures were gently washed five times (resuspension in ice-cold sterile water and centrifugation at 16,000 × *g* for 5 min). For anaerobiosis fixation, spores were washed with anaerobic reduced-PBS two times, followed by centrifugation prior fixation with 3% glutaraldehyde on a 0.1 M cacodylate buffer (pH 7.2). For spore purification, spores were gently loaded onto a 1.5 ml tube containing 300 μl of 45% Nycodenz solution and centrifuged for 40 min at 16,000 × *g*. After centrifugation, the supernatant was removed, and the spore pellet was washed five times. The spores were counted in a Neubauer chamber and concentration adjusted at 5 × 10^9^ spores/ml to store at −80°C until use.

### Transmission Electronic Microscopy (TEM)

Spores (2 × 10^8^) were fixed with 3% glutaraldehyde on a 0.1M cacodylate buffer (pH 7.2), incubated overnight at 4°C, and stained for 30 min with 1% tannic acid. Samples were further embedded in a Spurr resin (Paredes-Sabja et al., 2012). Thin sections of 90 nm were obtained with a microtome, placed on glow discharge carbon-coated grids and double lead stained with 2% uranyl acetate and lead citrate. Spores were analyzed with a Philips Tecnai 12 Bio Twin microscope at Unidad de Microscopía Avanzada in Pontificia Universidad Católica de Chile.

### Separation by Nycodenz-Gradient of *C. difficile* R20291 Spores

A Nycodenz-gradient was performed in which 64% Nycodenz was layered in the bottom followed by layers decreased in 1% until 56%. Spores (2.5 × 10^9^ spores) were gently added to the surface and centrifuged at 5,000 × *g* for 40 min in a swim bucket rotor. Two fractions were formed, which were recovered in aliquots of 1 ml by removing them gently from the surface of the Nycodenz gradient. Collected fractions were washed with 1 ml of sterile water and adjusted to a final concentration of 5 × 10^9^ spores/ml.

### Adherence of Fractionated *C. difficile* R20291 Spores

Caco-2 cells (ATCC, United States) were used for infection with separated spores at 2- and 8-days post confluence. Spores were added at a MOI of 10 and incubated for 3 h at 37°C under aerobic conditions with 5% CO2 (Mora-Uribe et al., 2016). Unbound spores were rinsed off with three washes of PBS. Unwashed wells were employed for total spore count. Caco-2 cells lysed in 80 μl 0.06% Triton X-100 for 30 min at 37°C. Cell-spore lysate were serially diluted, plated on BHIS-0.1% Sodium Taurocholate, and incubated in anaerobic conditions at 37°C for 48 h. The number of colony forming units (CFU) per ml was determined, and the percentage of adherence was calculated using the relation: (CFU ml^–1^/TOTAL CFU ml^–1^) × 100. The data represents the averages of the results of three independent experiments.

### Protein-SNAP Fusion Construction

According to the reference annotated genome of *C. difficile* strain R20291 (FN545816), genes and their coding regions used in this work were *cdeC* (CDR20291_0926), *cdeM* (CDR20291_1478), *bclA3* (CDR20291_3193), *cotA* (CDR20291_1511), *cdeB* (CDR20291_2642), *cotB* (CDR20291_1360), *cotE* (CDR20291_1282), *cotD* (CDR20291_0523) and their respective promotor region were independently cloned in plasmid pFT58. Briefly, promotor and coding region sequence were amplified with primers listed in Supplementary Table S1, and cloned between *Eco*RI/*Bam*HI sites in pFT58, which contains sequence for SNAP. Plasmid were stored in DH5α. Constructed plasmids are listed in Supplementary Table S2.

### Fluorescent Microscopy and Analysis of SNAP Fusion

Constructed plasmids (Supplementary Table S2) were conjugated into *C. difficile* R20291 using *E. coli* CA434 as the donor strain. Transconjugant *C. difficile* colonies were selected for resistance to 15 mg/ml thiamphenicol. *C. difficile* R20291 strains carrying SNAP fusion vectors were grown in BHIS – thiamphenicol, seeded in 70:30 at 1:100 dilution and incubated for 48 h at 37°C. A fraction of the plate was scrapped and resuspended in PBS-250 nM of SNAP-Cell^®^ Oregon Green^®^ (Invitrogen) and incubated for 30 min at 37°C (Cassona et al., 2016). Finally, labeled sample were pelleted and resuspended in PBS containing 10 ng/ul FM4-64 and 2.4 μM Hoechst, incubated at room temperature for 2 min and centrifugated 5 min at 6000 × *g*. The pellet was resuspended in 20 μl of PBS and 5 μl of the sample was mounted in coverslips with agarose pad and observed with BX53 Olympus fluorescence microscope. The fluorescence images were analyzed with ImageJ. At last, 200 spores were analyzed in three independent experiments.

## Results

### Thin and Thick Exosporium Spores Are Observed Simultaneously in Sporulating Cultures of *C. difficile*

We sought to determine whether the thick and thin exosporium morphotypes were formed simultaneously during spore formation. Sporulating cultures collected from 70:30 agar plates, were fixed under anaerobic conditions to assess whether the laminations of the spore-coat and exosporium thickness occurs during spore-development or after aerobic processing of the sporulating culture. Transmission electron micrographs from anaerobically fixed cultures show at early sporulation stage (1-day-old sporulation culture) several mother cells containing mature spores with both exosporium morphotypes (Figure 1A). We also observed that coat laminations can be clearly observed as soon as 24 h of sporulation (Figure 1B). At 1 day-old 70:30 sporulating cultures, percentages of thin and thick exosporium layer morphotypes are 84 and 16%, respectively (Figure 1C). In 5-days-old 70:30 sporulating cultures, the percentage of thin and thick exosporium morphotype spores was 76 and 24%, respectively (Figure 1B). Similar results were observed in TY agar media (Supplementary Figure S1). In TY medium, 1-day-old sporulating cultures revealed the presence of vegetative cells at different stages of the developmental process (i.e., asymmetrically divided cells, engulfed forespore and mature endospore) (Supplementary Figure S1A). Collectively, these results support the notion that during *C. difficile* sporulation spores with a thick or thin exosporium layer are formed simultaneously during sporulation.

### Enrichment of Polar Appendage and Thick Exosporium Morphotypes by Nycodenz Gradient of *C. difficile* Spores

**FIGURE 2:**
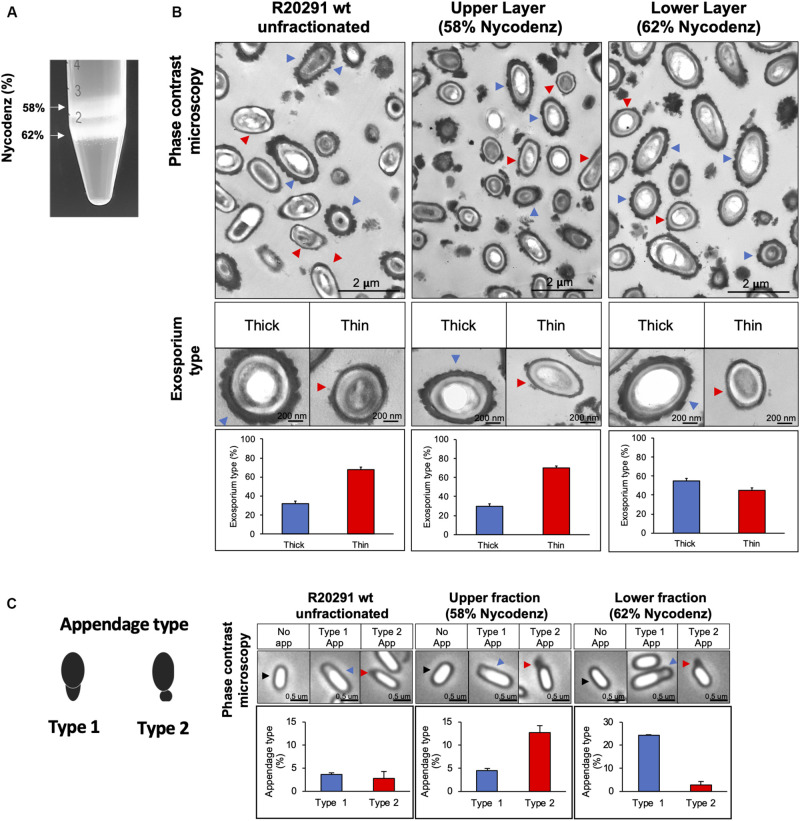
*C. difficile* R20291 spores separated in Nycodenz gradient. **(A)** Spores were separated with a Nycodenz gradient from 56 to 65% by centrifugation at 5,000 × *g* for 40 min. Two-fractions were obtained: fraction (58% Nycodenz) and lower fraction (62% Nycodenz). White arrows indicate both fractions. **(B)** Classification according to exosporium thickness of unfractionated, upper fraction, and lower fraction spores. Blue arrowheads indicate spores with a thick exosporium, and red arrowheads indicate spores with a thin exosporium. Bottom panel shows the percentage of each exosporium thickness (blue bar: thick exosporium, red bar: thin exosporium). **(C)** Appendage occurrence in unfractionated, upper fraction, and lower fraction spores observed by phase-contrast microscopy. The black arrowheads indicate spores with no appendages. Blue arrowheads correspond to type 1 appendage and red arrowheads to type 2 appendages. Percentage of appendage type is shown in the bottom panel (blue bar: type 1 appendages; red bar: type 2 appendages). At least 300 spores were counted from each gradient. The experiment was repeated three independent times.

The simultaneous appearance of both exosporium morphotypes during sporulation in different sporulating media (70:30 and TY) raised the question of whether *C. difficile* spores can be separated into two populations according to their exosporium thickness. Herein, we used a Nycodenz gradient to fractionate TY pure spores and observed two fractions of spores were the upper fraction stacked at 58% of Nycodenz and the lower fraction stacked at 62% of Nycodenz (Figure 2A). Transmission electron analysis revealed that while the upper fraction maintains the same proportion of thick (30% of spores) and thin (70% of spores) exosporium spores as unfractionated spores (Supplementary Figure S1), one-half of *C. difficile* spores in the lower fraction had thick exosporium (Figure 2B).

In the other hand, another characteristic that bifurcates the differentiation pathway in spore formation is the polar appendage, that can be present as a robust structure, a short appendage or be absent (Antunes et al., 2018). Additionally, short or absence of appendage are related to poor germination in response to taurocholate (Antunes et al., 2018). Phase contrast microscopy shows polar appendage in two different morphologies: appendage type 1, an exosporium prolongation in a polar region (Figure 2C), and appendage type 2, diffuse, round and loose prolongation of the exosporium. In unfractionated spores, only 6.4% of total spores exhibited appendage, being 3.6% type 1 and 2.8% type 2. Interestingly, appendage type 1 was enriched in the lower fraction (24.0% of spores) while appendage type 2 was enriched in the upper fraction (12.7% of spores). Collectively, these results provide evidence that thick exosporium and appendage type 1 can be enriched by a density gradient.

To assess whether these two spore-morphotypes could play a role in the adherence of spores to intestinal epithelial cells, we performed an infection assay of Caco-2 monolayers. Results show that adherence to epithelial cells is not different among fraction and is similar to unfractionated spores (Supplementary Figure S2). These results indicate that enrichment of appendages and thick-exosporium in the lower fraction does not affect spore-adherence to intestinal epithelial cells *in vitro*.

### Heterogenous Distribution of Spore Coat and Exosporium Proteins Detected by SNAP Translational Fusions in *C. difficile* R20291 Spores

Prior results demonstrated that intrinsic variability is operating at the exosporium level, as evidenced by appendage variability and exosporium layer variability (Pizarro-Guajardo et al., 2016a, b; Antunes et al., 2018). To identify the specific distribution of spore coat and exosporium proteins, translational SNAP fusions were used. *C. difficile* R20291 cultures take 48 h in 70:30 agar plate to render six morphological stages of sporulation (Supplementary Figure S3). 48-h sporulating culture incubated with SNAP reagent exhibited high heterogeneity in the pattern of distribution and intensity of fluorescence (Supplementary Figure S4). Statistical analysis of the fluorescence distribution revealed that none of these fusions followed a normal distribution (Shapiro-Wilk test, *P* < 0.0001) (Supplementary Figure S5), suggesting that two or more spore-populations might be present in each SNAP fusion proteins.

**FIGURE 3:**
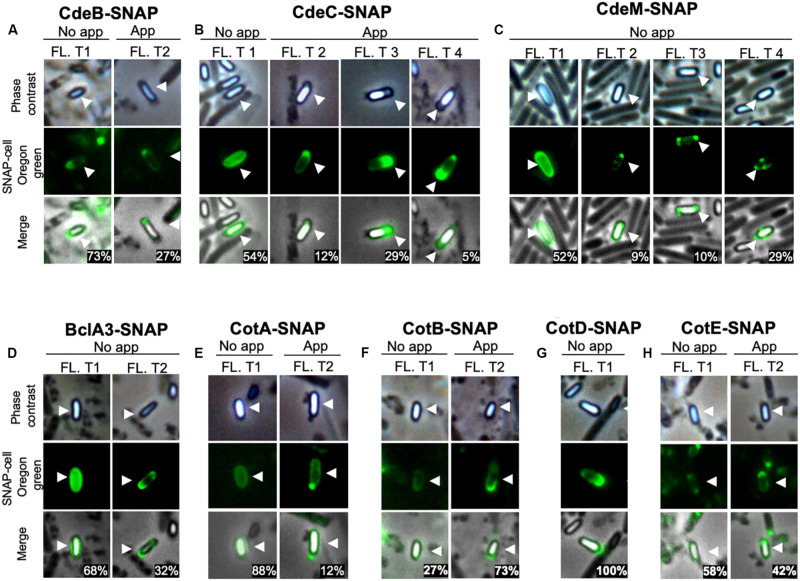
Fluorescence distribution of SNAP-spore-coat and -exosporium protein fusions in *C. difficile* spores. Localization of SNAP-fusions with exosporium proteins in *C. difficile* strain R20291. The strains expressing **(A)** CdeB-, **(B)** CdeC-, **(C)** CdeM-, **(D)** BclA3-, **(E)** CotA-, **(F)** CotB-, **(G)** CotD-, or **(H)** CotE-SNAP fusion were grown for 48 h in 70:30 agar plates and collected for labeling with Oregon green SNAP cell. Fl. t: fluorescence type. White arrows indicate the reference spore. The numbers in the merged images are the percentage of spores of each fluorescent type (Fl. T). Numbers in merge image indicate the mean of the relative fluorescence intensity. At least 400 spores were analyzed for each SNAP-fusion.

Patterns of fluorescence distribution shows of fluorescence shows that several fluorescence types can be found when coat and exosporium SNAP fusion proteins are examined (Figure 3), in appendage and appendage-free spores in unfractionated spore population. Specifically, we observed that spores carrying CdeB-SNAP fusion exhibit only polar fluorescence signal, despite presence or absence of polar appendage (Figure 3A). The cysteine rich protein CdeC-SNAP fusion shows homogeneous fluorescence around the spore when the appendage is absent in 54% of fluorescent spores, whereas positive-appendage spores had three types of fluorescence distribution at the poles (Figure 3B). Notably, regardless of the absence of polar appendage in spores carrying CdeM-SNAP fusion, only half of fluorescent spores (52%) exhibited a homogeneous fluorescence distribution around the spore; while other fluorescence types were evidenced at one, both poles or at one pole-and-sides of the spore (Figure 3C). Homogeneous fluorescence around spores was observed in 68% of appendage-negative spores carrying BclA3-SNAP fusion, while the rest exhibit polar-and-side fluorescence (Figure 3D). Among spore coat protein-fusions, we observed that the spores carrying CotA-SNAP fusion had a regular fluorescence signal around the spore, and a small fraction had a strong fluorescence intensity in the spore pole where a type 2 appendage appears (Figure 3E). By contrast, spores carrying CotB-, CotD-, and CotE-SNAP fusions exhibited pole-located fluorescence signal (Figures 3F–H).

Since translational fusions of SNAP protein were cloned into plasmid pFT48, which yields 4-10 copy number per cell (Pereira et al., 2013; Ransom et al., 2015), we assessed whether the overexpression of exosporium and spore coat proteins fused to the SNAP reporter protein would affect the formation of appendage (Supplementary Figure S6). Nearly 6% of the spores carrying the empty vector exhibited appendage. No significant increase in the percentage of appendage was evidenced in spores carrying CdeB (8% of total spores). However, spores carrying CdeC-, CotB-, and CotE-SNAP fusion had an increased appendage-positive spores (Supplementary Figure S6). Expression of CdeM-, BclA3-, CotA-, and CotD-SNAP fusions led to completely lost of appendage (Supplementary Figure S6). Collectively, these results demonstrate that multicopy expression of SNAP fusion proteins differentially affects appendage formation in *C. difficile* spores.

### Effect of Overexpression of the Exosporium Proteins CdeC, and CdeM on the Ultrastucture of R20291 Spore

**FIGURE 4:**
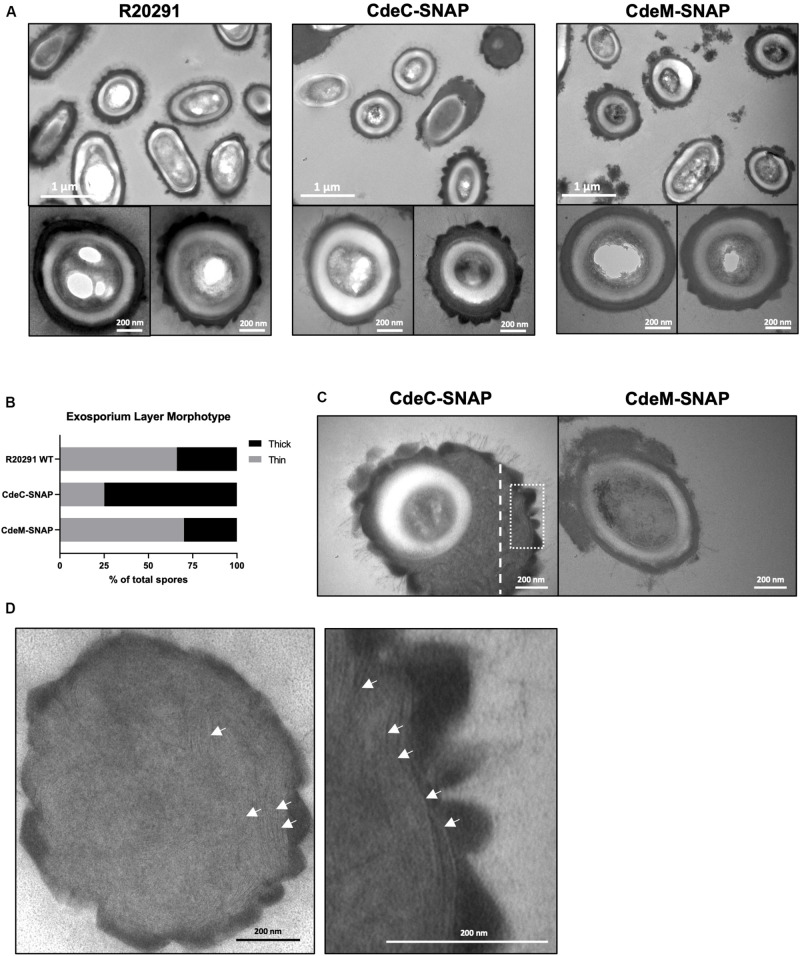
CdeC-SNAP fusions affects the exosporium ultrastructure of *C. difficile* R20291 spores. **(A)** Purified spores of the CdeC-, CdeM-SNAP fusions were analyzed by TEM and the formation of exosporium morphotypes was evaluated. Diffuse loose material surrounding spores expressing CdeM-SNAP fusion is evidenced. Bar scale: 1 μm and 200 nm. **(B)** Exosporium morphotypes were quantified in each SNAP fusion. **(C)** Spores carrying CdeC-SNAP spores exhibiting an accumulation of electron-dense material around the spore in one or two poles. Spores carrying CdeM-SNAP exhibit a disorganized exosporium and accumulation of material. **(D)** The first panel is representative of the thin section of the thick exosporium layer of CdeC-SNAP spore depicted with dotted white line in Panel C. The second panel is a magnified section of the TE micrograph of the dotted white square line of CdeC-SNAP spores in Panel C. White arrows denote the laminations in the thick exosporium layer.

Since exosporium protein CdeC and CdeM are key proteins for the proper assembly of the spore outer layers and appendage (Barra-Carrasco et al., 2013; Calderón-Romero et al., 2018), we evaluate if the extrachromosomal overexpression of translational SNAP fusions of these exosporium proteins (CdeC and CdeM) affect ultrastructural features of *C. difficile* spores. Both exosporium morphotypes (thin and thick) were observed in *C. difficile* R20291 carrying empty vector (Figure 4A); thin exosporium is the most abundant morphotype (Figure 4A), while the thick morphotype is present in 30% of spores, consistent with previous reports (Pizarro-Guajardo et al., 2016b). Interestingly, overexpression of CdeC resulted in nearly 75% of the spores with a thick exosporium layer (Figures 4A,B). CdeC-SNAP fusion also resulted in an amorphous accumulation of electron-dense material in the spore surface with bumps and hair-like projections (Figure 4C); these spores correspond to nearly 28% of the analyzed spore sample (*n* = 100 spores). Expression of CdeM-SNAP fusion did not impact the proportion of exosporium morphotypes, however, *C. difficile* strain expressing CdeM-SNAP fusion produced spores with a disorganized, diffuse and loose external material (Figure 4C). In some cases, this material seems to be loosely attached to the polar pole of the spore (Figure 4C). A detail analysis of the thick exosporium layer of CdeC-SNAP carrying spores revealed the presence of disorganized laminations; TEM micrographs of a thin section of the amorphous thick exosporium layer of CdeC-SNAP carrying spores shows the presence of poorly organized laminations (Figure 4D). Magnification of the exosporium layer shows that these are different from those previously reported in the spore coat (Figure 4D). Collectively, these results suggest that episomal expression of CdeC-SNAP fusion, but not CdeM-SNAP, affects the abundance and morphology of thick exosporium morphotype.

## Discussion

*C. difficile* spores are metabolically dormant life forms that are able to resist antibiotic exposure and act as transmission vectors, disseminating disease. The outermost layer of *C. difficile* spores is thought to act as the site of contact during the first stages of infection. Most epidemically relevant strains have an exosporium with hair-like projections that builds on top of a thin layer of electron dense material surrounding the spore coat; alternatively, these projections may also build on top of a thicker layer of electron dense material that surrounds the spore coats (Pizarro-Guajardo et al., 2016b). In this work, we provide more insight into the variability of these two types of exosporium layer of *C. difficile* spores.

A first finding of this work was that the different spore-morphotypes are formed simultaneously during sporulation. The simultaneous formation of both exosporium morphotypes (thin and thick) observed by transmission electron micrographs of sporulating cultures of R20291 in 70:30 as well as in TY agar plates suggests that the mechanism that drives each morphotype occurs independent of the culture conditions and in a subset of sporulating cells (Figure 1). These observations also show that final morphotype is independent of sporulation timing (Figure 1). Though it is intriguing that the small fraction of thick exosporium spores is maintained in different sporulating medium conditions and exhibited an increase in aged sporulating cultures, suggesting that environmental conditions and/or additional unidentified stimuli might be implicated in thickness of the exosporium layer. However, several questions arise; there is no genetic evidence of such a regulatory circuit that might regulate thickness of the outer layer of *C. difficile* spores. Although, potential candidates include the excision of the skin element in the mother cell by the recombinase gene *spoIVCA* which leads to active SigK and spores of *C. difficile* 630 strain with an electron dense exosporium layer (Serrano et al., 2016). Further studies to identify and characterize the mechanisms that regulate the formation of thin and/or thick exosporium morphotypes are underway.

Several tools have been developed to track down proteins in anaerobic species such as *C. difficile* (Ransom et al., 2015; Cassona et al., 2016). Here we provide evidence that, there is great variability in the expression of SNAP reporter fusions with exosporium and spore coat proteins of *C. difficile*. We observed high variability in the fluorescence intensity and also in the distribution pattern of SNAP-fusion proteins. First, some spores exhibited a low fluorescence intensity, which could be attributed to poor folding of the SNAP fluorescent fusion protein or to low expression levels during spore-development. In accordance to these observations, Imamura et al. (2011), observed that not all of the spores expressing GFP- fusion proteins of the outer-most layer of *B. subtilis* spores yielded detectable fluorescence (Imamura et al., 2011), although they did not quantify the fluorescence intensity. We also observed that the fluorescence distribution does not follow a normal distribution, and in most cases exhibited a skewed distribution, indicating that there might be subpopulation of spores with different spore surface protein compositions. Supporting this notion, we observed that the fluorescent distribution of CdeC-, CdeM-, BclA3-, and CotA-SNAP fusions exhibited two distinctive patterns of fluorescence (Figure 3): (i) a homogenous distribution surrounding the spore; and (ii) a defined pattern at one or both poles of the spore. Antunes et al. (2018) reported similar variability in the fluorescence pattern of SNAP-translational fusion with the exosporium protein, CdeM (Antunes et al., 2018). These differences in the fluorescence patterns could be attributed to the differences in the hierarchical network of genetic dependencies, thus, slight increase on the levels of a specific spore-coat and/or exosporium protein could result in changes in the levels and distribution of other spore constituents. Such an example of the hierarchical network for some spore coat and exosporium proteins was recently reported by Calderón-Romero et al. (2018) provide evidence that the absence of CdeC or CdeM affect the presence of BclA3, CdeB, CotA and CotB in strain 630 (Calderón-Romero et al., 2018). While, absence of CdeM affects the presence of CdeC but not vice versa in a 630 background (Calderón-Romero et al., 2018). A major question that the differential distribution of the fluorescent intensity of exosporium protein-SNAP fusions is attributed to the exosporium type (i.e., thin and thick). Indeed, based on our previous findings that thick and thin exosporium spores also differ in the thickness of the underlying spore coat layers, it is plausible that these two types of spores might also have different levels of spore coat proteins. Studies to address these questions will provide insight into the mechanisms underlying the variability of the exosporium layer of *C. difficile* spores.

We were able to separate R20291 spores into two spore populations; one less dense fraction with similar abundance of thick exosporium layer spores and appendage-positive spores as unfractionated spores; and a denser fraction, enriched in thick exosporium layer spores and polar appendages. These observations are in agreement with the enrichment of appendage positive spores of strain 630 through density gradient (Antunes et al., 2018). Although, our results suggest that both phenotypes (i.e., thickness of the exosporium layer and the presence/absence of polar appendage) become enriched in the same gradient fraction, it is likely that the mechanism driving appendage formation and thick exosporium layer might be different. For example, absence of CdeM affects the thickness of the exosporium layer but not appendages presence (Antunes et al., 2018; Calderón-Romero et al., 2018). However, Antunes et al. (2018) results indicate that spores enriched in appendages obtained from higher density fraction (i.e., 50-55%) exhibit oligomers of CdeC and CdeM, whereas those from the lower density gradient (i.e., 45%) have monomeric CdeC and CdeM in the spore coat/exosporium extracts, supporting the notion that both proteins might be implicated in exosporium differentiation. In our experimental conditions, we observed no difference in the adherence of spores from the upper and lower fractions to monolayers of epithelial Caco-2 cells. By contrast, Antunes et al. (2018), demonstrated that 630 spores enriched in appendage by density gradient germinated faster than fractions of spores with no appendage (Antunes et al., 2018). The exact role of these phenotypes in *C. difficile* pathogenesis is unclear and require further research.

Recent work has demonstrated that cysteine-rich proteins are responsible for the correct formation of the exosporium layer of *C. difficile* spores in several genetic backgrounds (Barra-Carrasco et al., 2013; Antunes et al., 2018; Calderón-Romero et al., 2018). In R20291 and 630 strain, inactivation of *cdeC* leads to spores with an aberrantly assembled exosporium layer, which is an accumulation of diffuse material in the exosporium that lacks the electron-dense material, as well as alterations in the thickness of the spore coat and exosporium layer (Barra-Carrasco et al., 2013; Calderón-Romero et al., 2018). By contrast, in 630 strain, inactivation of *cdeM* only leads to loss of the exosporium layer without affecting the underlying layers (Calderón-Romero et al., 2018). In this context, our results demonstrate that the multicopy expression of CdeC- and CdeM-SNAP fusion proteins lead to different effects on the surface of *C. difficile* spores (Figure 4). Multicopy expression of CdeC-SNAP led to an increase in the ratio of thick-exosporium spores upon as well as an increase in spores with an amorphously thick exosporium layer (Figure 4). It was also noteworthy to observe that the amorphous exosporium layer of these spores had electron-dense material deposited on the bumps formed and the presence of the typical hair-like projections observed in wild-type spores. Underlying this electron dense material is a series of disorganized laminations that are likely to be due to the over-expression of CdeC-SNAP, suggesting that CdeC might be forming part of these structural features. These observations indicates that CdeC-SNAP fusion is functional, and that CdeC, is able to drive the formation of thick-exosporium layer and presumably of disorganized laminations. On the other hand, the CdeM-SNAP fusion, yielded spores with a similar ratio of thin/thick exosporium layer as spores carrying vector control, but with immunofluorescence signal through the entire spore as well as in one or both poles of the spores (Figure 4). Multi-copy expression of CdeM-SNAP had no effect on the proportion of thin/thick exosporium ratio but led to an increased number of spores with a diffuse loose material partially surrounding the spore, resembling the phenotype of a *cdeM* mutant, suggesting that CdeM-SNAP fusion has a negative-dominant phenotypic effect on the exosporium layer. It is noteworthy that CdeC seems to be buried under CdeM, which would be in agreement with CdeC acting as a driver in the thickening of the exosporium layer (Antunes et al., 2018). Altogether these results contribute to understand the variability in the outermost layer of *C. difficile* spores and raise new questions about the underlying mechanisms of assembly and variability of this layer.

## Data Availability Statement

All datasets presented in this study are included in the article/Supplementary Material.

## Author Contributions

AR-R, MP-G, PC-R, and DP-S contributed to the conception and design of the study. AR-R, MP-G, and PC-R executed the experiments. MP-G and PC-R performed the statistical analysis. MP-G and DP-S wrote the first draft of the manuscript. AR-R, DP-S, and MP-G wrote sections of the manuscript. All authors contributed to manuscript revision, read and approved the submitted version.

## Conflict of Interest

The authors declare that the research was conducted in the absence of any commercial or financial relationships that could be construed as a potential conflict of interest.

## Supplementary Material

FIGURE S1 Transmission electron micrographs of 1-day- and 5-day-old sporulating cultures in TY agar plates. **(A)** Sporulating cultures in TY medium harvested after 1 day (left panel) and 5 days (right panel) of growth in TY medium. Cultures were fixed under anaerobic conditions prior to processing for transmission electron micrograph analysis. White arrow: thin exosporium spore, Black arrow: thick exosporium spore. Bar scale: 2 μm. **(B)** Ultrastructural analysis of the exosporium layer. Thin and thick exosporium morphotype spores evidenced at day 1 (left panel) and day 5 (right panel). Scale bar: 200 nm. **(C)** Percentage of thin and thick exosporium at day 1 (gray bar) and day 5 (black bar). Bar indicates standard error obtained with three different batch of culture fixed, processed and analyzed separately. *N* = 50 spores. *P*-value indicated.

FIGURE S2 Adherence of nycodenz-separated *C. difficile* R20291 spores to intestinal epithelial Caco-2 cell *in vitro*. **(A)** Monolayers of undifferentiated 2-day-old Caco-2 and, **(B)**, differentiated 8-day-old Caco-2 cells were infected at a MOI of 10 with *C. difficile* R20291 unfractionated, upper and lower fractions spores for 3 h at 37°C. Unbound spores were removed by rinsing with PBS. Adherence was quantified as described in the Method section. Graphs represent the average of three independent experiments, error bars represent standard errors of the means. n.s.: no significance.

FIGURE S3 Sporulation of *C. difficile* R20291. **(A)** Dynamics of sporulation. Different stages of the sporulation process: stage 1, vegetative cell; stage 2, asymmetric division with septum; stage 3, curvature in septum membrane; stage 4, conformed forespore; stage 5, pre-spore found as a bright structure; and stage 6, spore released. Membrane (FM4-64; red) and DNA (Hoechst; blue) stains are shown. White arrowhead indicates the cell or spore in the corresponding stage. Sporulation process is represented: mother cell membrane lined in blue, spore inner membrane in red and spore external layers in green. **(B)** Quantification of total cell or spore of each stage in time (18, 24, and 48 h). Total cells for the quantification >1800.

FIGURE S4 SNAP fusions proteins in coat and exosporium of *C. difficile* R20291 spores. Phase-contrast microscopy and fluorescence microscopy of *C. difficile* R20291 containing SNAP fusion, cultured by 48 h in 70:30 agar plate and incubated with 250 nM of SNAP cell Oregon green (New England Biolabs^®^). White arrow indicates high fluorescence spores. Scale bar: 10 μm.

FIGURE S5 Frequency distribution of SNAP proteins fusions of *C. difficile* R20291 spores. *C-difficile* containing each SNAP fusion was independently incubated with 250 nM for 48 h with SNAP cell Oregon green (New England Biolabs^®^). **(A)** CdeB-SNAP; **(B)** CotA-SNAP; **(C)** CdeC-SNAP; **(D)** CotB-SNAP; **(E)** CdeM-SNAP: **(F)** CotD-SNAP; **(G)** BclA3-SNAP; **(H)** CotE-SNAP. *n* > 300. Shapiro-Wilk test was used and all sporulating cultures carrying SNAP fusions of exosporium and spore-coat proteins had *P* < 0,0001.

FIGURE S6 Presence or absence of appendage of *C. difficile* R20291 spores with SNAP fusions. Percentage of *C. difficile* R20291 spores with empty vector and SNAP fusions are shown. The classification was made observing the spores in contrast phase microscopy of spores that carrying the SNAP fusions.
