# Supplementary material for: Characterization of Exosporium Layer Variability of Clostridioides difficile Spores in the Epidemically Relevant Strain R20291
Source: Front Microbiol. 2020 Jul 2;11:1345. doi: 10.3389/fmicb.2020.01345 (PMC7343902; doi:10.3389/fmicb.2020.01345)
Supplement: Supplementary file 7 [file Data_Sheet_1.PDF]

Table S1. Primers used in this work to create SNAP fusions.

Gene Locus[a]	Primer name	Primer sequence[b]	Position[c]
cdeC (CDR20291_0926)	P499 FP-cdeC-0926-EcoRI	GACGAATTCAATGAAATACGGGAGACCGTGTCTGG	-318 to -293
	P500 RP-cdeC-0926-BamHI	GACGGATCCTCTGTGGCAACTTGGCTTTCC	+1195 to +1218
cdeM (CDR20291_1478)	P562 FP-cdeM-1478-EcoRI	GACGAATTCCTGAATATGAAATAAACAACAGTTTATGTC	- 199 to -169
	P563 RP-cdeM-1478-BamHI	GACGGATCCTTTTCTACAGCAGTTACAATTACATTTATG	+466 to 492
bclA3 (CDR20291_3193)	P508 FP-bclA3-3193-EcoRI	GACGAATTCGCTAAAGAGTACGGGCTGATTG	-1524 to -1503
	P509 RP-bclA3-3193-BamHI	GACGGATCCATTTATTGCAATTCCTGCACTTG	+2011 to +2034
cotA (CDR20291_1511)	P511 FP-cotA-1511-EcoRI	GACGAATTCTACTTCCTGATGTTGGTGTTAATATGCCA	-484 to -456
	P512 RP-cotA-1511-BamHI	GACGGATCCTGCAATATAATCTATAGAATCTACACATACAAAG	+ 891 to +924
cdeB (CDR20291_2642)	P597 FP-cdeB-2642-EcoRI	TATATGAATTCCATACTCAAATTCTTCATCATC	-422 to -401
	P598 RP-cdeB-2642-BamHI	GACGGATCCGTTAAGATTTCCTGCTTTATTAG	+641 to +663
cotB (CDR20291_1360)	P599 FP-cotB-1360-EcoRI	TAATGAATTCCTTGAAATTGTTTGCATACTTAATT	-563 to -539
	P600 RP-cotB-1360-BamHI	GACGGATCCCATGTTTTTATAACTCTCCAATATTC	+886 to +912
cotE (CDR20291_1282)	P601 FP-cotE-1282-EcoRI	TATCAGAATTCCATGGAGATAACTAAAATTCTTAG	-268 to -245
	P602 RP-cotE-1282-BamHI	GACGGATCCGAATTGCCCATAAATACCTTCAAGTTC	+2101 to +2128
cotD (CDR20291_0523)	P603 FP-cotD-0523-EcoRI	CAGAAAGAATTCGCACAGAAAAAAGAGTAGG	-978 to -950
	P604 RP-cotD-0523-BamH1	TACGGATCCGAACTGATGCTTACACTC	+550 to +570

[a] Data base: EnsemblGenomes Gene, NCBI FN545816_R20291.

b Restriction site is underlined.

[c] The nucleotide position number begins from the first codon and refers to the relevant position within the respective gene sequence.

Table S2. Plasmids and strains used in this work

Plasmid or strain name	Description	Reference
pFT58	Derived from pMTL84121, contains SNAP sequence lacking start codon, between BamHI/HindIII sites.	(Pereira et al. 2013)
pPCR15	CdeC-SNAP translational fusion. A 1553 bp PCR fragment amplified with P499/P500, cloned in BamHI/EcoRI sites of pFT58.	This work
pPCR16	CdeM-SNAP translational fusion. A 1131 bp PCR fragment amplified with P562/P563, cloned in BamHI/EcoRI sites of pFT58.	This work
pPCR10	BclA3-SNAP translational fusion. A 3683 bp PCR fragment amplified with P508/P509, cloned in BamHI/EcoRI sites of pFT58.	This work
pPCR11	CotA-SNAP translational fusion. A 1107 bp PCR fragment amplified with P511/P512, cloned in BamHI/EcoRI sites of pFT58.	This work
pARR3	CdeB-SNAP translational fusion. A 1090 bp PCR fragment amplified with P597/P598, cloned in BamHI/EcoRI sites of pFT58.	This work
pARR2	CotB-SNAP translational fusion. A 1480 bp PCR fragment amplified with P599/P600, cloned in BamHI/EcoRI sites of pFT58.	This work
pARR6	CotE-SNAP translational fusion. A 2400 bp PCR fragment amplified with P601/P602, cloned in BamHI/EcoRI sites of pFT58.	This work
pARR5	CotD-SNAP translational fusion. A 1554 pb PCR fragment amplified with P603/P604, cloned in BamHI/EcoRI sites of pFT58.	This work
**Strain**	**Characteristics**	**Reference**
C. difficile R20201	Ribotype 027, epidemically relevant strain	(McEllistrem et al. 2005)
C. difficile R20291 (pPCR15)	*C. difficile* R20291 carrying *cdeC*-SNAP fusions	This work
C. difficile R20291 (pPCR16)	*C. difficile* R20291 carrying *cdeM*-SNAP fusions	This work
C. difficile R20291 (pPCR11)	*C. difficile* R20291 carrying *cotA*-SNAP fusions	This work
C. difficile R20291 (pPCR10)	*C. difficile* R20291 carrying *bclA3*-SNAP fusions	This work
C. difficile R20291 (pARR6)	*C. difficile* R20291 carrying *cotE*-SNAP fusions	This work
C. difficile R20291 (pARR5)	*C. difficile* R20291 carrying *cotD*-SNAP fusions	This work
C. difficile R20291 (pARR2)	*C. difficile* R20291 carrying *cotB*-SNAP fusions	This work
C. difficile R20291 (pARR3)	*C. difficile* R20291 carrying *cdeB*-SNAP fusions	This work
C. difficile R20291 (pFT58)	*C. difficile* R20291 carrying empty vector	(Pereira et al. 2013)
